# Maintained renin–angiotensin–aldosterone system inhibitor therapy with sodium zirconium cyclosilicate following a hyperkalaemia episode: a multicountry cohort study

**DOI:** 10.1093/ckj/sfae083

**Published:** 2024-03-25

**Authors:** Anjay Rastogi, Charles V Pollack, Ignacio José Sánchez Lázaro, Eva Lesén, Matthew Arnold, Stefan Franzén, Alaster Allum, Ignacio Hernández, Toyoaki Murohara, Eiichiro Kanda

**Affiliations:** Department of Medicine, David Geffen School of Medicine, University of California Los Angeles, Los Angeles, CA, USA; Department of Emergency Medicine, University of Mississippi School of Medicine, Jackson, MS, USA; Cardiology Department, Hospital Universitari i Politècnic La Fe, Valencia, Spain; BioPharmaceuticals Medical CVRM Evidence, AstraZeneca, Gothenburg, Sweden; Real World Science and Digital, AstraZeneca, Cambridge, UK; Medical & Payer Evidence Statistics, AstraZeneca, Gothenburg, Sweden; BioPharmaceuticals Medical CVRM, AstraZeneca, Cambridge, UK; Atrys Health, Madrid, Spain; Department of Cardiology, Nagoya University Graduate School of Medicine, Nagoya, Japan; Department of Medical Science, Kawasaki Medical School, Okayama, Japan

**Keywords:** chronic kidney disease, heart failure, hyperkalaemia, renin–angiotensin–aldosterone system inhibitor, sodium zirconium cyclosilicate

## Abstract

**Background:**

This observational cohort study compared the likelihood of maintained (stabilized/up-titrated) renin–angiotensin–aldosterone system inhibitor (RAASi) therapy at 6 months following hyperkalaemia in patients with chronic kidney disease (CKD) and/or heart failure (HF) from the USA, Japan and Spain who received sodium zirconium cyclosilicate (SZC) for at least 120 days, relative to those with no prescription for a potassium (K^+^) binder.

**Methods:**

Using health registers and hospital medical records, patients with CKD and/or HF receiving RAASi therapy who experienced a hyperkalaemia episode were identified. Propensity score (PS) matching (1:4) was applied to balance the SZC cohort to the no K^+^ binder cohort on baseline characteristics. Logistic regression analysis was performed to compare the odds of maintained RAASi therapy at 6 months in the SZC versus no K^+^ binder cohorts.

**Results:**

The PS-matched SZC cohort included 565 (USA), 776 (Japan) and 56 (Spain) patients; the no K^+^ binder cohort included 2068, 2629 and 203 patients, respectively. At 6 months, 68.9% (USA), 79.9% (Japan) and 69.6% (Spain) in the SZC cohorts versus 53.1% (USA), 56.0% (Japan) and 48.3% (Spain) in the no K^+^ binder cohorts had maintained RAASi therapy. Meta-analysed across countries, the odds ratio of maintained RAASi therapy in the SZC cohort versus no K^+^ binder cohort was 2.56 (95% confidence interval 1.92–3.41; *P* < .0001).

**Conclusions:**

In routine clinical practice across three countries, patients treated with SZC were substantially more likely to maintain guideline-concordant RAASi therapy at 6 months following hyperkalaemia relative to patients with no K^+^ binder treatment.

## Graphical Abstract

**Graphical Abstract:**
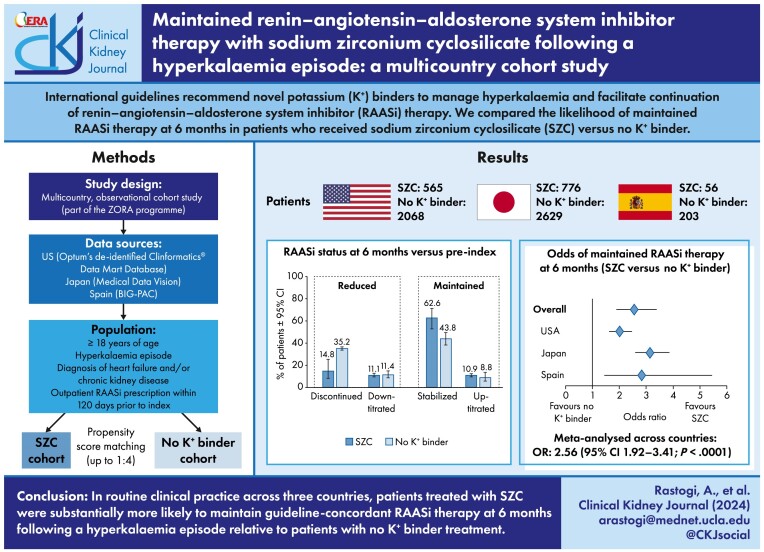

KEY LEARNING POINTS **What was known:**

- Renin–angiotensin–aldosterone system inhibitors (RAASi) are foundational therapy in the management of chronic kidney disease (CKD) and heart failure (HF) but increase the risk of hyperkalaemia.
- Clinical guidelines recommend novel potassium binders to manage hyperkalaemia and facilitate continuation and optimization of RAASi therapy.

**This study adds:**

- In routine clinical practice across three countries, the likelihood of maintained (stabilized/up-titrated) RAASi therapy at 6 months following a hyperkalaemia episode in patients with CKD and/or HF was substantially higher in those receiving sodium zirconium cyclosilicate, relative to no potassium binder treatment [odds ratio 2.56 (95% confidence interval 1.92–3.41)].

**Potential impact:**

- These findings demonstrate the potential value of novel potassium binder therapy to facilitate maintained and guideline-concordant RAASi therapy following a hyperkalaemia episode to help achieve optimal treatment outcomes in patients with CKD and/or HF.

## INTRODUCTION

Because of their demonstrated cardio- and renoprotective effects in reducing the risk of kidney failure and cardiovascular mortality and morbidity, renin–angiotensin–aldosterone system inhibitors (RAASi) are foundational therapy in the management of chronic kidney disease (CKD) and heart failure (HF) [1–3]. International evidence-based guidelines recommend RAASi therapy at the maximal tolerated dose to optimize treatment benefits in patients with CKD and/or HF [1–3].

However, RAASi therapy increases the risk of hyperkalaemia, which can lead to cardiac arrhythmias, cardiac arrest and death if left untreated [4–6]. The increased risk of hyperkalaemia with RAASi therapies results from their pharmacodynamic potassium (K^+^)-sparing properties via inhibition of the production of or increased resistance to aldosterone as well as through a reduction of renal K^+^ excretion [7–9]. In a previous meta-analysis, the risk of developing hyperkalaemia was twice as high among patients receiving RAASi compared with those not receiving RAASi [10]. Despite the strong evidence of beneficial effects with RAASi, the risk of hyperkalaemia is perceived as a barrier to achieving guideline-directed therapy with RAASi. Multiple studies demonstrate that in routine clinical practice, RAASi therapy is often compromised in patients with hyperkalaemia [11–15]. Compared with maintained or up-titrated RAASi dosing, hyperkalaemia-related discontinuation or down-titration of RAASi dosing is associated with a higher risk of cardiac and renal adverse events and mortality [14, 16].

Current international guidelines state that hyperkalaemia should not be a barrier to RAASi optimization and specifically recommend novel K^+^ binders to manage hyperkalaemia and facilitate continuation of RAASi therapy, with a matching consensus from practicing clinicians [1–3, 17, 18]. Two K^+^ binders are referenced: sodium zirconium cyclosilicate (SZC) and patiromer, which were approved in 2018 and 2015, respectively, for the treatment of adults with hyperkalaemia [19, 20]. SZC has demonstrated beneficial effects in optimizing RAASi therapy. In an open-label, single-arm, phase 3 trial, SZC maintained normokalaemia without substantial changes to RAASi for up to 12 months; of the 483 RAASi users at baseline, 87% continued their RAASi or had their dose increased [21]. Furthermore, a US observational study found that nearly 80% of patients with hyperkalaemia who initiated outpatient treatment with SZC in routine clinical practice maintained or up-titrated their RAASi therapy [22]. However, direct comparative studies of SZC versus no K^+^ binder treatment on the ability to maintain RAASi therapy have not been performed previously.

The aim of this multicountry, observational cohort study was to compare the likelihood of maintained (stabilized or up-titrated) RAASi therapy at 6 months following a hyperkalaemia episode among patients with CKD and/or HF treated with SZC for at least 120 days relative to those receiving no K^+^ binder treatment. This study is part of ZORA, an observational study programme investigating the management and consequences of hyperkalaemia in patients with CKD and/or HF in routine clinical practice [14].

## MATERIALS AND METHODS

### Data sources

This observational cohort study is based on longitudinal data collected retrospectively from health claims and hospital medical records in the USA (Optum's Clinformatics Data Mart), Japan [Medical Data Vision (MDV)] and Spain (BIG-PAC). The Optum Clinformatics Data Mart is a de‐identified administrative health database comprising claims data from recipients of commercial health insurance and Medicare Advantage plans across the USA. The database includes detailed enrolment information, diagnoses and procedures recorded in inpatient and outpatient care settings, prescription drugs and some laboratory results [23]. The MDV database captures healthcare data for ≈38 million patients from hospitals across Japan, including information on diagnoses and procedures and prescriptions recorded in inpatient and outpatient care settings. Laboratory test results are captured from a subset of hospitals [24]. The BIG-PAC administrative database includes anonymized electronic medical records data for nearly 2 million patients from primary and secondary care within the Spanish national health system across seven regions [25].

The study period varied among data sources, starting when SZC became available in each respective country and ending at the last date of available data in each data source: July 2019–December 2022 for the USA, May 2020–December 2022 for Japan and June 2021–December 2022 for Spain.

The US data source included de-identified data in compliance with the Health Insurance Portability and Accountability Act requirements; neither informed consent nor institutional review board approvals were required. According to the Japanese Ethical Guidelines for Medical and Health Research Involving Human Subjects, ethical approval and informed consent do not apply to the use of de-identified secondary data. In Spain, the study was approved by the Ethical Committee (Comité de Ética de Investigación con Medicamentos del Consorci Sanitari de Terrassa; 02-23-399-061), with a waiver of requirement for informed consent.

### Study population

**Figure 1:**
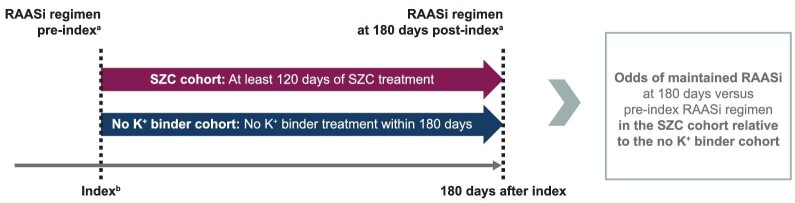
Study design. ^a^Defined based on the most recently filled prescriptions of RAASi medications (ACEi, ARB, ARNi, MRA) within the preceding 120 days. ^b^Index date is the initiation of SZC (SZC cohort) or hyperkalaemia diagnosis/discharge date (no K^+^ binder cohort).

The study included two cohorts: SZC and no K^+^ binder (Fig. 1). The SZC cohort included patients who were issued or dispensed a prescription of SZC and had evidence of at least 120 days of continuous SZC treatment, allowing a gap in supply of no more than 7 days. The requirement of at least 120 days of continuous SZC treatment was included to identify patients with longer-term SZC treatment. Due to hyperkalaemia being the sole indication for SZC, as well as the known under-recording of hyperkalaemia diagnoses in routine practice and the extent of missing data on laboratory K^+^ values in some of the data sources, feasibility analyses were performed to determine whether the characteristics of SZC patients with versus without a recorded hyperkalaemia diagnosis in the preceding 30 days were similar enough to combine into one cohort. The index date in the SZC cohort was the date of the first SZC prescription of the continuous treatment episode.

The no K^+^ binder cohort included patients with a recorded inpatient or outpatient hyperkalaemia episode (International Classification of Diseases, Tenth Revision, code E87.5) and without a prescription for any type of K^+^ binder (SZC, patiromer, sodium polystyrene sulfonate or calcium polystyrene sulfonate) during the 180 days of follow-up. The index date in this cohort was defined as the discharge date of an inpatient episode or date of outpatient care visit with a recorded hyperkalaemia diagnosis.

Patients included in either cohort were also required to be ≥18 years of age and have a diagnosis of HF and/or CKD, but not to be on haemodialysis during the baseline period (12 months prior to the index date), and to have an outpatient prescription for at least one type of RAASi medication within 120 days prior to the index date. RAASi classes included angiotensin-converting enzyme inhibitors (ACEi), angiotensin receptor blockers (ARB), angiotensin receptor–neprilysin inhibitors (ARNi) and mineralocorticoid receptor antagonists (MRA). At least 12 months of available look-back for baseline data prior to the index date were required for inclusion, as well as at least 180 days of available follow-up time after the index date to allow for outcomes assessment (Fig. 1).

All potential index dates for each patient were screened for eligibility. If a patient had more than one eligible index date for the same cohort at different time points, the index date for inclusion in that cohort was selected at random. If a patient had eligible index dates for both cohorts at different time points, the patient was included in both cohorts (at the corresponding index date).

Propensity score (PS) matching was applied to balance the SZC cohort with the no K^+^ binder cohort, with the following covariates as candidates for inclusion: baseline demographics (age and sex), comorbidities based on recorded diagnoses (CKD by stage, arrhythmia, HF, coronary heart disease, diabetes and proteinuria), comedications [use of RAASi and by level of guideline target dose, alpha blockers, beta blockers, beta agonists, cardiac glycosides, calcium channel blockers, calcium gluconate, diuretics (any), loop diuretics, thiazide diuretics, insulin, sodium bicarbonate, non-steroidal anti-inflammatory drugs and sodium–glucose co-transporter-2 inhibitors], hyperkalaemia [index severity (mild/moderate/severe) and recurrent/new onset], history of K^+^ binder use and estimated glomerular filtration rate. Logistic regression modelling was used for PS estimation, with a calliper of 0.10 standard deviations (SDs) of the logit PS. A PS matching ratio of up to 1:4 (SZC:no K^+^ binder) was used in each dataset. Covariates with a standardized mean difference (SMD) <10% after matching were considered balanced.

### Outcome definition

The primary outcome was the proportion of patients who maintained RAASi therapy at 180 days post-index. The definition of maintained versus reduced RAASi therapy was based on which RAASi classes (ACEi, ARB, ARNi and MRA) and the doses for which the patients had prescriptions pre-index (using a 120-day look-back period) versus at 180 days post-index (similarly using a 120-day look-back period). Maintained RAASi therapy was defined as having post-index prescriptions for at least the same number of RAASi classes as pre-index; this category encompassed stabilized RAASi (use of the same number of RAASi classes and doses) or up-titrated (use of additional RAASi classes and/or higher doses). Reduced RAASi therapy was defined as RAASi therapy that was discontinued (no filled prescription for any RAASi class) or down-titrated (use of fewer RAASi classes or when the dose of at least one pre-index RAASi class was reduced by ≥25% post-index).

A sensitivity analysis was performed in which the definition of RAASi therapy did not include MRA (i.e. only ACEi, ARB and ARNi).

### Statistical analyses

Study outcomes were analysed in the PS-matched cohorts. The proportions of patients in the SZC and in the no K^+^ binder cohorts who up-titrated, stabilized, down-titrated or discontinued RAASi therapy at 180 days post-index versus pre-index were calculated and *P*-values for differences between groups were calculated from χ^2^ tests. The proportions were meta-analysed across countries using a random effects model on logit transformed proportions.

Logistic regression analysis was performed to compare the odds of maintained RAASi therapy in the SZC versus no K^+^ binder cohorts. Covariates that were not sufficiently balanced between the cohorts after matching (SMD >10%) were considered for inclusion in the multivariable model, while ensuring a minimum of 10 outcome events per covariate. The odds ratios (ORs) and associated 95% confidence intervals (CIs) were meta-analysed across countries using a random effects model.

Subgroup analyses were performed for patients with CKD, HF, CKD + HF and diabetes. For each of these subgroup analyses, a new PS matching was performed using the cases and controls in the corresponding subgroup (data not shown).

## RESULTS

### Study sample

Patient attrition for each cohort is summarized in Supplementary data, Fig. S1. A total of 582, 888 and 104 patients from the USA, Japan and Spain, respectively, met the study eligibility criteria for the SZC cohorts prior to PS matching. Of the SZC-treated patients in the USA and Spain, 58.8% and 51.0%, respectively, did not have a preceding hyperkalaemia diagnosis recorded within the 30 days prior to index. In both cohorts, the characteristics of patients with versus without a recorded preceding hyperkalaemia diagnosis were deemed sufficiently similar to be combined into a common SZC cohort (Supplementary data, Table S1). In Japan, 97.9% of SZC-treated patients had a documented preceding hyperkalaemia diagnosis and all were included regardless of the specific documentation of a hyperkalaemia diagnosis.

A total of 102 537, 22 771 and 2274 patients from the USA, Japan, and Spain, respectively, met the study eligibility criteria for the no K^+^ binder cohort prior to PS matching. Patient characteristics of each cohort, prior to PS matching, are presented in Supplementary data, Table S2.

Following PS matching, the SZC cohorts consisted of 565, 776 and 56 patients from the USA, Japan and Spain, respectively, and the no K^+^ binder cohorts consisted of 2068, 2629 and 203 patients, respectively. The covariates included in the PS matching are listed in Supplementary data, Fig. S2; all covariates in the USA and Japan had an absolute SMD <10% after matching, while some covariates remained unbalanced in Spain. The PS distributions before and after matching are shown in Supplementary data, Fig. S3.

Baseline patient demographics and characteristics of the PS-matched SZC and no K^+^ binder cohorts are shown in Table 1. In the USA, the mean age was 72.1 and 72.4 years in the SZC and no K^+^ binder cohorts, 44.1% and 44.6% were female, 96.5% and 96.6% had CKD and 34.7% and 34.2% had HF, respectively. In Japan, the mean age was 75.8 and 76.2 years in the SZC and no K^+^ binder cohorts, 33.3% and 33.7% were female, 81.3% and 85.2% had CKD and 73.7% and 74.6% had HF, respectively. In Spain, the mean age was 71.8 and 69.4 years in the SZC and no K^+^ binder cohorts, 41.1% and 41.4% were female, 75.0% and 75.9% had CKD and 32.1% and 34.5% had HF, respectively.

**Table 1:** Patient characteristics of the PS-matched SZC and no K^+^ binder cohorts at baseline.

	USA		Japan		Spain	
Characteristics	SZC (*n* = 565)	No K^+^ binder (*n* = 2068)	SZC (*n* = 776)	No K^+^ binder (*n* = 2629)	SZC (*n* = 56)	No K^+^ binder (*n* = 203)
Age, years, mean ± SD	72.1 ± 10.2	72.4 ± 9.8	75.8 ± 10.9	76.2 ± 10.3	71.8 ± 8.3	69.4 ± 11.1
Sex (female), *n* (%)	249 (44.1)	923 (44.6)	258 (33.3)	886 (33.7)	23 (41.1)	84 (41.4)
HK severity at index, *n* (%)^a^,^b^						
Mild	30 (28.0)	114 (27.6)	21 (28.0)	69 (40.8)	15 (26.8)	55 (27.1)
Moderate	44 (41.1)	160 (38.7)	30 (40.0)	57 (33.7)	31 (55.4)	116 (57.1)
Severe	33 (30.8)	139 (33.7)	24 (32.0)	43 (25.4)	10 (17.9)	32 (15.8)
Missing	458 (81.1)	1655 (80.0)	701 (90.3)	2460 (93.6)	0 (0)	0 (0)
CKD, *n* (%)	545 (96.5)	1997 (96.6)	631 (81.3)	2239 (85.2)	42 (75.0)	154 (75.9)
CKD stage, *n* (%)^a^						
CKD3	247 (47.2)	892 (46.2)	40 (18.7)	108 (15.2)	25 (61.0)	103 (74.1)
CKD4	217 (41.5)	820 (42.5)	75 (35.0)	270 (38.0)	13 (31.7)	33 (23.7)
CKD5	59 (11.3)	219 (11.3)	99 (46.3)	333 (46.8)	3 (7.3)	3 (2.2)
Missing	27 (4.8)	88 (4.3)	417 (53.7)	1528 (58.1)	1 (1.8)	15 (7.4)
Heart failure, *n* (%)	196 (34.7)	708 (34.2)	572 (73.7)	1960 (74.6)	18 (32.1)	70 (34.5)
Diabetes, *n* (%)	446 (78.9)	1657 (80.1)	725 (93.4)	2467 (93.8)	34 (60.7)	123 (60.6)
Recurrent or new-onset hyperkalaemia, *n* (%)						
New-onset	43 (7.6)	153 (7.4)	148 (19.1)	280 (10.7)	3 (5.4)	2 (1.0)
Recurrent	522 (92.4)	1915 (92.6)	628 (80.9)	2349 (89.4)	53 (94.6)	201 (99.0)
K^+^ binder prescription in the 12 months before index, *n* (%)	302 (53.5)	979 (47.3)	572 (73.7)	2151 (81.8)	30 (53.6)	54 (26.6)

a Proportions are calculated excluding missing data.

b Mild, moderate and severe hyperkalaemia equate to K^+^ values of 5–5.49, 5.5–5.99 and ≥6.0 mmol/l, respectively.

### RAASi treatment

The proportions of patients using each RAASi class pre-index and at 180 days post-index are shown in Table 2. Although data were analysed descriptively, some trends were apparent. The proportions of patients using ARNi were similar or increased from pre- to post-index in the SZC cohort and generally decreased in the no K^+^ binder cohort. The proportions of patients using ACEi, ARB and MRA generally decreased in both cohorts from pre- to post-index, although reductions were numerically larger in the no K^+^ binder cohort.

**Table 2:** Patients with prescriptions for each RAASi class pre-index and at 180 days post-index (PS matched).

	USA		Japan		Spain	
Characteristic	SZC (*n* = 565)	No K^+^ binder (*n* = 2068)	SZC (*n* = 776)	No K^+^ binder (*n* = 2629)	SZC (*n* = 56)	No K^+^ binder (*n* = 203)
Pre-index						
ACEi	249 (44.1)	913 (44.2)	117 (15.1)	391 (14.9)	25 (44.6)	96 (47.3)
ARB	265 (46.9)	998 (48.3)	610 (78.6)	2069 (78.7)	28 (50.0)	99 (48.8)
ARNi	40 (7.1)	136 (6.6)	57 (7.4)	190 (7.2)	4 (7.1)	15 (7.4)
MRA	78 (13.8)	259 (12.5)	145 (18.7)	467 (17.8)	11 (19.6)	41 (20.2)
Post-index						
ACEi	189 (33.5)	540 (26.1)	95 (12.2)	231 (8.8)	18 (32.1)	65 (32.0)
ARB	212 (37.5)	656 (31.7)	515 (66.4)	1261 (48.0)	25 (44.6)	61 (30.0)
ARNi	41 (7.3)	104 (5.0)	110 (14.2)	196 (7.5)	4 (7.1)	10 (4.9)
MRA	57 (10.1)	129 (6.2)	129 (16.6)	270 (10.3)	10 (17.9)	21 (10.3)

Values are presented as *n* (%).

The proportions of patients who discontinued, down-titrated, stabilized or up-titrated their RAASi therapy in each country are presented in Table 3. The proportions who remained on any RAASi therapy (i.e. had not discontinued) at 180 days were consistently higher in the SZC cohorts than in the no K^+^ binder cohorts (USA: 80.2% versus 64.8%, *P* < .0001; Japan: 90.7% versus 64.8%, *P* < .0001; Spain: 82.1% versus 64.0%, *P* = .0102).

**Table 3:** Proportions of patients who discontinued, down-titrated, stabilized and up-titrated their RAASi therapy post-index versus pre-index in the USA, Japan and Spain.

Overall	SZC	No K^+^ binder	*P*-value
USA	*n* = 565	*n* = 2068	
Discontinued	112 (19.8)	727 (35.2)	<.0001
Down-titrated	64 (11.3)	243 (11.8)	.8386
Stabilized	327 (57.9)	976 (47.2)	<.0001
Up-titrated	62 (11.0)	122 (5.9)	<.0001
Japan	*n* = 776	*n* = 2629	
Discontinued	72 (9.3)	926 (35.2)	<.0001
Down-titrated	84 (10.8)	232 (8.8)	.1059
Stabilized	536 (69.1)	1283 (48.8)	<.0001
Up-titrated	84 (10.8)	188 (7.2)	.0012
Spain	*n* = 56	*n* = 203	
Discontinued	10 (17.9)	73 (36.0)	.0102
Down-titrated	7 (12.5)	32 (15.8)	.5455
Stabilized	33 (58.9)	63 (31.0)	.0001
Up-titrated	6 (10.7)	35 (17.2)	.2362

Values are presented as *n* (%).

*P*-values for differences between the SZC cohort versus the no K^+^ binder cohort in the proportions of patients who discontinued, down-titrated, stabilized and up-titrated their RAASi therapy at 180 days post-index versus pre-index were calculated using the χ^2^ test.

**Figure 2:**
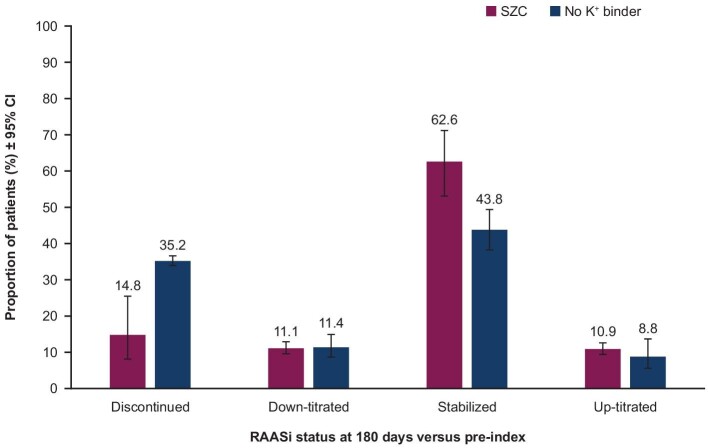
Meta-analysed proportions of patients who discontinued, down-titrated, stabilized and up-titrated their RAASi therapy post-index versus pre-index. The proportions were meta-analysed across countries using a random effects model on logit transformed proportions. *I*^2^ = 93.3% (SZC discontinued), 0.0% (no K^+^ binder discontinued), 0.0% (SZC down-titrated), 88.3% (no K^+^ binder down-titrated), 89.1% (SZC stabilized), 91.3% (no K^+^ binder stabilized), 0.0% (SZC up-titrated) and 94.0% (no K^+^ binder up-titrated).

Overall, the meta-analysed proportions of patients who had discontinued their RAASi therapy were lower in the SZC cohort than in the no K^+^ binder cohort (14.8% versus 35.2%) (Fig. 2). The proportions of patients who down-titrated RAASi therapy were similar in the SZC versus no K^+^ binder cohorts (11.1% versus 11.4%). Furthermore, higher proportions of patients in the SZC cohort than in the no K^+^ binder cohort had stabilized (62.6% versus 43.8%) and up-titrated (10.9% versus 8.8%) their RAASi therapy (Fig. 2).

**Figure 3:**
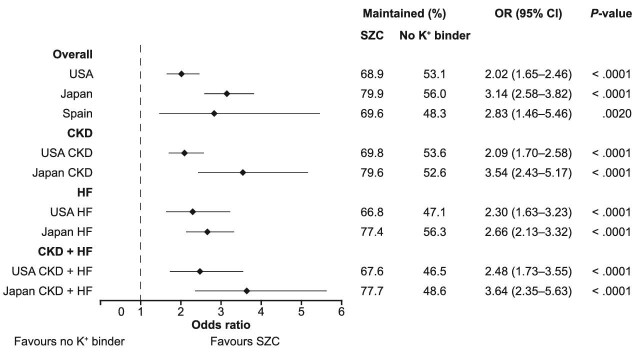
Odds of maintained RAASi therapy in the SZC versus no K^+^ binder cohorts, by country and subgroup. A logistic regression analysis was performed to compare the odds of maintained (stabilized or up-titrated) RAASi therapy in the SZC versus no K^+^ binder cohorts. The logistic regression model for Spain was adjusted for age, due to the observed imbalance after PS matching. Subgroup analyses for CKD, HF and CKD + HF were not performed in Spain due to the limited sample size.

At 180 days post-index, 68.9% (USA), 79.9% (Japan) and 69.6% (Spain) of patients in the SZC cohorts versus 53.1% (USA), 56.0% (Japan) and 48.3% (Spain) of patients in the no K^+^ binder cohorts maintained (stabilized or up-titrated) their pre-index RAASi therapy. In the USA, Japan and Spain, respectively, the ORs for maintained RAASi therapy were 2.02 (95% CI 1.65–2.46), 3.14 (95% CI 2.58–3.82) and 2.83 (95% CI 1.46–5.46) for those treated with SZC relative to no K^+^ binder treatment (Fig. 3). The logistic regression model for Spain was adjusted for age, due to the observed imbalance after PS matching. Meta-analysed across countries, the odds of maintained RAASi therapy were 2.5 times higher in the SZC versus the no K^+^ binder cohort [OR 2.56 (95% CI 1.92–3.41); *P* < .0001; *I*^2^ = 68.8%].

The sensitivity analysis, in which the definition of maintained versus reduced RAASi therapy did not include MRA, was performed in the USA and Japan; Spain was not included due to the limited sample size. Results from the sensitivity analysis supported the primary analysis (Supplementary data, Table S3).

### Subgroup analyses

Subgroup analyses on patients with CKD, HF, CKD + HF and diabetes were performed in the cohorts from the USA and Japan; Spain was not included due to the limited sample size. The baseline patient demographics and characteristics of the subgroups are shown in Supplementary data, Tables S4–S6. The proportions of patients who discontinued, down-titrated, stabilized and up-titrated their RAASi therapy at 180 days post-index versus pre-index are shown for the CKD, HF and CKD + HF subgroups in Supplementary data, Tables S7–S9. Across patient subgroups and countries, the results of the subgroup analysis were consistent with the primary analysis. In patients with CKD, the proportions who remained on any RAASi at 180 days were consistently higher in the SZC cohorts than in the no K^+^ binder cohorts (USA: 80.6% versus 65.8%, *P* < .0001; Japan: 88.6% versus 59.8%, *P* < 0.0001). A similar pattern was observed in patients with HF (USA: 78.8% versus 65.7%, *P* = .0008; Japan: 90.4% versus 66.7%, *P* < .0001). Results in the diabetes subgroup were consistent with the other patient subgroups (data not shown).

The results of the country-level logistic regression analyses for the odds of maintained pre-index RAASi therapy with SZC versus no K^+^ binder among the patient subgroups are shown in Fig. 3. The meta-analysed ORs for maintained RAASi therapy for those treated with SZC relative to no K^+^ binder treatment were 2.65 (95% CI 1.62–4.34; *P* < .0001; *I*^2^ = 81.0%) in the CKD subgroup, 2.54 (95% CI 2.08–3.10; *P* < .0001; *I*^2^ = 9.4%) in the HF subgroup and 2.94 (95% CI 2.01–4.31; *P* < .0001; *I*^2^ = 45.3%) in the CKD + HF subgroup.

## DISCUSSION

Despite recommendations from international guidelines [1–3], RAASi therapy in clinical practice is commonly down-titrated or discontinued following a hyperkalaemia episode, and the RAASi reduction tends to persist over time [14]. The present multicountry observational cohort study compared the odds of maintained RAASi therapy at 6 months following a hyperkalaemia episode among patients with CKD and/or HF treated with SZC for at least 120 days relative to no K^+^ binder treatment.

This analysis shows that treatment with SZC more than doubled the odds of maintaining (i.e. stabilized or up-titrated) RAASi therapy following hyperkalaemia relative to no K^+^ binder treatment. Furthermore, the findings were consistent across three different countries. Another key finding is the lower occurrence of RAASi discontinuation associated with SZC treatment, with fewer than half as many patients (14.8% versus 35.2%) discontinuing RAASi therapy in the SZC cohort than the no K^+^ binder cohort. The results on RAASi therapy patterns observed here correspond with a recent observational, non-comparative study that included US patients treated with SZC [22].

The findings of increased odds of maintained RAASi therapy and a lower occurrence of RAASi discontinuation with SZC treatment have clinical importance, given that hyperkalaemia-related discontinuation or down-titration of RAASi therapy is associated with a higher risk of cardiac and renal adverse events and mortality when compared with continued RAASi therapy [14, 16, 26]. In one analysis, the risk of adverse cardiorenal outcomes (HF emergency visit, HF hospitalization or progression to end-stage kidney disease) at 6 months in US patients was 17.5% in those who discontinued RAASi therapy and 10.6% in those who maintained or up-titrated; the corresponding risk in Japan was 19.7% and 15.1%, respectively [14]. An increased risk of all-cause mortality associated with RAASi discontinuation was similarly observed [14]. Overall, these findings support the importance of novel K^+^ binder treatment in maintaining cardio- and renoprotective RAASi therapy following an episode of hyperkalaemia.

Results from the sensitivity analysis, in which the definition of RAASi therapy did not include MRA, are in alignment with the main findings. In clinical practice, MRA use has tended to be reserved for patients with more severe HF [27], although recent guidelines now recommend use of an MRA in all patients with HF with reduced left ventricular ejection fraction, regardless of severity [1, 17].

CKD and HF are commonly co-occurring diseases [28]. Accordingly, we performed subgroup analyses to assess the odds of maintained RAASi therapy among patients with CKD, HF or CKD + HF in the USA and Japan (subgroup analyses were not performed in Spain due to the limited sample size). The increased odds of maintained RAASi therapy associated with SZC versus no K^+^ binder treatment observed in the primary analysis were also consistently observed across the patient subgroups, indicating that findings are robust regardless of patient population. These findings align with international guidelines for patients with CKD and with HF, which recommend novel K^+^ binder treatment to maintain RAASi therapy following hyperkalaemia [3, 17].

A strength of this analysis is the inclusion of patients from three different countries. Patients from the USA, Japan and Spain had differences in their baseline demographics, clinical characteristics and RAASi use and have access to different healthcare systems, thus representing geographically diverse populations. For example, the US cohorts had a higher occurrence of CKD than the Japan and Spain cohorts (≈97% versus 75–85%), while the Japan cohorts had a higher occurrence of HF (≈75% versus 32–35%) than the USA and Spain cohorts. Furthermore, the Japan cohorts had a greater occurrence of previous K^+^ binder use and a greater use of ARB than the US and Spain cohorts. Data sources differed in terms of availability of laboratory data, and the presence of a hyperkalaemia diagnosis, rather than laboratory evidence of elevated K^+^, was used to identify patients with hyperkalaemia. Furthermore, the proportion of patients in the SZC cohorts with a recorded preceding hyperkalaemia diagnosis differed between countries. In Japan, 98% of the SZC cohort had a preceding hyperkalaemia diagnosis, while such a diagnosis was not recorded in approximately half of patients in the USA and Spain. However, a feasibility assessment deemed that the characteristics of SZC patients with versus without a preceding hyperkalaemia diagnosis in these two countries were sufficiently similar to be combined into a common SZC cohort. Importantly, despite these differences between populations and data sources, significantly greater odds of maintaining RAASi therapy with SZC versus no K^+^ binder treatment were consistently observed across the three countries, demonstrating the robustness and generalizability of these findings.

This analysis has limitations. All patients in each cohort were required to have at least 180 days of available follow-up time after the index date, which imposed some magnitude of immortal time bias. However, this affected the SZC cohort and the no K^+^ binder cohort equally, by potentially excluding the sickest patients from both cohorts, and was essential for the assessment of the RAASi outcomes, comparing both use and dose levels across the four RAASi classes rather than simply overall RAASi discontinuation. The SZC cohort was required to have at least 120 days of continuous SZC treatment, with the rationale to include patients with longer-term SZC treatment. Findings may therefore have limited generalizability for patients with shorter SZC use.

The primary outcome was defined based on data on issued or dispensed drug prescriptions collected via health claims and hospital medical records, which may have a lower specificity compared with similar endpoints on drug utilization collected prospectively in clinical trials. Furthermore, some baseline clinical characteristics (such as hyperkalaemia severity at the index date and CKD stage) were missing in some of the datasets; however, this is an inherent limitation of observational studies. Sensitivity and subgroup analyses were not possible in the Spain cohorts due to the limited sample size; meta-analyses in subgroups were therefore restricted to data from the US and Japan cohorts. In addition, the meta-analyses on the logit transformed proportions and on the ORs are not expected to correspond exactly due to the mathematical nature of the log odds function. Despite balancing cohorts on potential baseline confounders using PS matching, some risk of residual confounding may remain even after adjustment. Finally, SZC was the only K^+^ binder assessed in the current analysis, so the findings may not be generalizable to other K^+^ binders. Future research is warranted to investigate the association between the duration of SZC treatment and the likelihood of maintained RAASi therapy across various subgroups of patients.

## CONCLUSION

In contemporary routine clinical practice across three countries, patients treated with SZC were substantially more likely to have maintained (stabilized/up-titrated) RAASi therapy at 6 months following a hyperkalaemia episode relative to patients with no K^+^ binder treatment. These findings are clinically important, as they demonstrate the potential of SZC treatment to facilitate maintained and guideline-concordant RAASi therapy following an episode of hyperkalaemia, which is associated with a reduced risk of cardiorenal adverse events and mortality in patients with CKD or HF.

## Supplementary Material

sfae083_Supplemental_File

## Data Availability

Data underlying the findings described in this article may be obtained from the corresponding author upon reasonable request, in accordance with AstraZeneca's data sharing policy described at https://astrazenecagrouptrials.pharmacm.com/ST/Submission/Disclosure. However, restrictions apply to these data, which were used under license and/or specific approval for the current study and are not publicly available.
