# Supplementary material for: Maintained renin–angiotensin–aldosterone system inhibitor therapy with sodium zirconium cyclosilicate following a hyperkalaemia episode: a multicountry cohort study
Source: Clin Kidney J. 2024 Mar 25;17(5):sfae083. doi: 10.1093/ckj/sfae083 (PMC11062025; doi:10.1093/ckj/sfae083)
Supplement: sfae083_Supplemental_File [file sfae083_supplemental_file.docx]

# Supplementary material for:

**Maintained renin–angiotensin–aldosterone system inhibitor therapy with sodium zirconium cyclosilicate following a hyperkalaemia episode: a multicountry cohort study**

Anjay Rastogi^1^, Charles V. Pollack Jr.^2^, Ignacio José Sánchez Lázaro^3^, Eva Lesén^4^, Matthew Arnold^5^, Stefan Franzén^6^, Alaster Allum^7^, Ignacio Hernández^8^, Toyoaki Murohara^9^, and Eiichiro Kanda^10^

^1^Department of Medicine, David Geffen School of Medicine at the University of California Los Angeles, Los Angeles, CA, USA; ^2^Department of Emergency Medicine, University of Mississippi School of Medicine, Jackson, MS, USA; ^3^Cardiology Department, Hospital Universitari i Politècnic La Fe, Valencia, Spain; ^4^BioPharmaceuticals Medical CVRM Evidence, AstraZeneca, Gothenburg, Sweden; ^5^Real World Science and Digital, AstraZeneca, Cambridge, UK; ^6^Medical & Payer Evidence Statistics, AstraZeneca, Gothenburg, Sweden; ^7^BioPharmaceuticals Medical CVRM, AstraZeneca, Cambridge, UK; ^8^Atrys Health, Madrid, Spain; ^9^Department of Cardiology, Nagoya University Graduate School of Medicine, Nagoya, Japan; ^10^Department of Medical Science, Kawasaki Medical School, Okayama, Japan

Supplementary Table S1: Characteristics of SZC-treated patients with versus without a recorded preceding HK diagnosis.

| Characteristic | USA | | Spain | |
| --- | --- | --- | --- | --- |
|  | SZC with a HK diagnosis^a^  (*n* = 240) | SZC without a HK diagnosis^a^ (*n* = 342) | SZC with a HK diagnosis^a^  (*n* = 51) | SZC without a HK diagnosis^a^ (*n* = 53) |
| Age, years, mean ± SD | 72.8 ± 9.6 | 71.4 ± 10.6 | 73.5 ± 9.7 | 70.4 ± 7.4 |
| Sex, female, *n* (%) | 108 (45.0) | 144 (42.1) | 19 (37.3) | 27 (50.9) |
| CKD, *n* (%) | 234 (97.5) | 328 (95.9) | 43 (84.3) | 43 (81.1) |
| CKD stage, *n* (%)^b^ |  |  |  |  |
| CKD3 | 103 (45.8) | 147 (46.7) | 20 (46.5) | 24 (57.1) |
| CKD4 | 100 (44.4) | 131 (41.6) | 12 (27.9) | 13 (31.0) |
| CKD5 | 22 (9.8) | 37 (11.7) | 11 (25.6) | 5 (11.9) |
| Missing | 11 (4.6) | 16 (4.7) | 0 (0) | 1 (1.8) |
| Heart failure, *n* (%) | 98 (40.8) | 103 (30.1) | 20 (39.2) | 18 (34.0) |
| Diabetes, *n* (%) | 191 (79.6) | 270 (79.0) | 30 (58.8) | 31 (58.5) |
| ACEi use, *n* (%) | 252 (43.3) | 145 (42.4) | 22 (43.1) | 21 (39.6) |
| ARB use, *n* (%) | 277 (47.6) | 162 (47.4) | 29 (56.9) | 25 (47.2) |
| ARNi use, *n* (%) | 43 (7.4) | 28 (8.2) | 3 (5.9) | 1 (1.9) |
| MRA use, *n* (%) | 81 (13.9) | 48 (14.0) | 15 (29.4) | 11 (20.8) |

^a^Defined as HK diagnosis within 30 days prior to and including index date (first SZC prescription fill).
^b^Proportions are calculated excluding missing data.
ACEi, angiotensin-converting enzyme inhibitor; ARB, angiotensin receptor blocker; ARNi, angiotensin receptor-neprilysin inhibitor; CKD, chronic kidney disease; HK, hyperkalaemia; MRA, mineralocorticoid receptor antagonist; SD, standard deviation; SZC, sodium zirconium cyclosilicate.

Supplementary Table S2: Patient characteristics of the SZC and no K^+^ binder cohorts at baseline before propensity score matching.

| Characteristic | USA | | Japan | | Spain | |
| --- | --- | --- | --- | --- | --- | --- |
|  | SZC  (*n* = 582) | No K^+^ binder  (*n* = 102 537) | SZC  (*n* = 888) | No K^+^ binder (*n* = 22 771) | SZC (*n* = 104) | No K^+^ binder (*n* = 2274) |
| Age, years, mean ± SD | 72.0 ± 10.2 | 74.0 ± 9.7 | 75.8 ± 10.7 | 75.6 ± 11.7 | 71.9 ± 8.7 | 74.7 ± 9.4 |
| Sex, female, *n* (%) | 252 (43.3) | 51 571 (50.3) | 289 (32.6) | 8785 (38.6) | 46 (44.2) | 984 (43.3) |
| HK severity at index, *n* (%)^a,b^ |  |  |  |  |  |  |
| Mild | 30 (27.5) | 6530 (38.8) | 21 (20.4) | 267 (60.0) | 21 (20.2) | 702 (34.0) |
| Moderate | 46 (42.2) | 6771 (40.2) | 45 (43.7) | 115 (25.8) | 58 (55.8) | 1060 (51.3) |
| Severe | 33 (30.3) | 3537 (21.0) | 37 (35.9) | 63 (14.2) | 25 (24.0) | 304 (14.7) |
| Missing | 473 (81.3) | 85 699 (83.6) | 785 (88.4) | 22 326 (98.0) | 0 (0) | 0 (0) |
| CKD, *n* (%) | 562 (96.6) | 83 289 (81.2) | 730 (82.2) | 17 105 (75.1) | 86 (82.7) | 1608 (70.7) |
| CKD stage, *n* (%)^a^ |  |  |  |  |  |  |
| CKD3 | 250 (46.3) | 49 448 (69.9) | 44 (17.9) | 968 (19.2) | 44 (51.8) | 943 (65.9) |
| CKD4 | 231 (42.8) | 16 388 (23.2) | 95 (38.6) | 1355 (26.8) | 25 (29.4) | 396 (27.7) |
| CKD5 | 59 (10.9) | 4893 (6.9) | 107 (43.5) | 2724 (54.0) | 16 (18.8) | 91 (6.4) |
| Missing | 27 (4.6) | 14 378 (14.0) | 484 (54.5) | 12 058 (53.0) | 1 (1.0) | 178 (7.8) |
| Heart failure, *n* (%) | 201 (34.5) | 50 876 (49.6) | 643 (72.4) | 17 825 (78.3) | 38 (36.5) | 629 (27.7) |
| Diabetes, *n* (%) | 461 (79.2) | 69 157 (67.5) | 833 (93.8) | 20 161 (88.5) | 61 (58.7) | 1081 (47.5) |
| K^+^ binder prescription in the  12 months before index, *n* (%) | 319 (54.8) | 5742 (5.6) | 593 (66.8) | 11 437 (50.2) | 57 (54.8) | 118 (5.2) |
| ACEi use, *n* (%) | 252 (43.3) | 53 554 (52.2) | 128 (14.4) | 3747 (16.5) | 43 (41.3) | 1017 (44.7) |
| ARB use, *n* (%) | 277 (47.6) | 39 101 (38.1) | 706 (79.5) | 16 853 (74.0) | 54 (51.9) | 1178 (51.8) |
| ARNi use, *n* (%) | 43 (7.4) | 4350 (4.2) | 70 (7.9) | 825 (3.6) | 4 (3.8) | 203 (8.9) |
| MRA use, *n* (%) | 81 (13.9) | 20 441 (19.9) | 159 (17.9) | 5075 (22.3) | 26 (25.0) | 598 (26.3) |

^a^Proportions are calculated excluding missing data.
^b^Mild, moderate and severe HK equate to K^+^ values of 5–5.49, 5.5–5.99, and ≥6.0 mmol/l, respectively.
ACEi, angiotensin-converting enzyme inhibitor; ARB, angiotensin receptor blocker; ARNi, angiotensin receptor-neprilysin inhibitor; CKD, chronic kidney disease; HK, hyperkalaemia; K^+^, potassium; MRA, mineralocorticoid receptor antagonist; SD, standard deviation; SZC, sodium zirconium cyclosilicate.

Supplementary Table S3: Sensitivity analysis (excluding MRA from the definition of RAASi) on the proportions of patients who discontinued, down‑titrated, stabilized and up-titrated their RAASi therapy post-index versus pre‑index (USA and Japan).

1. **Overall**

|  | SZC | No K^+^ binder |
| --- | --- | --- |
| **USA** | *n* = 548 | *n* = 2002 |
| Discontinued | 113 (20.6) | 722 (36.1) |
| Down-titrated | 48 (8.8) | 164 (8.2) |
| Stabilized | 329 (60.0) | 999 (49.9) |
| Up-titrated | 58 (10.6) | 117 (5.8) |
| **Japan** | *n* = 749 | *n* = 2500 |
| Discontinued | 71 (9.5) | 882 (35.3) |
| Down-titrated | 53 (7.1) | 157 (6.3) |
| Stabilized | 552 (73.7) | 1315 (52.6) |
| Up-titrated | 73 (9.8) | 146 (5.8) |

1. **CKD**

|  | **SZC** | **No K^+^ binder** |
| --- | --- | --- |
| **USA** | *n* = 515 | *n* = 1877 |
| Discontinued | 106 (20.6) | 661 (35.2) |
| Down-titrated | 40 (7.8) | 164 (8.7) |
| Stabilized | 311 (60.4) | 937 (49.9) |
| Up-titrated | 58 (11.3) | 115 (6.1) |
| **Japan** | *n* = 206 | *n* = 672 |
| Discontinued | 24 (11.7) | 270 (40.2) |
| Down-titrated | 9 (4.4) | 40 (6.0) |
| Stabilized | 151 (73.3) | 319 (47.5) |
| Up-titrated | 22 (10.7) | 43 (6.4) |

1. **HF**

|  | **SZC** | **No K^+^ binder** |
| --- | --- | --- |
| **USA** | *n* = 185 | *n* = 667 |
| Discontinued | 45 (24.3) | 234 (35.1) |
| Down-titrated | 13 (7.0) | 80 (12.0) |
| Stabilized | 102 (55.1) | 307 (46.0) |
| Up-titrated | 25 (13.5) | 46 (6.9) |
| **Japan** | *n* = 545 | *n* = 1772 |
| Discontinued | 52 (9.5) | 602 (34.0) |
| Down-titrated | 45 (8.3) | 122 (6.9) |
| Stabilized | 386 (70.8) | 927 (52.3) |
| Up-titrated | 62 (11.4) | 121 (6.8) |

1. **CKD + HF**

|  | **SZC** | **No K^+^ binder** |
| --- | --- | --- |
| **USA** | *n* = 166 | *n* = 594 |
| Discontinued | 44 (26.5) | 231 (38.9) |
| Down-titrated | 7 (4.2) | 63 (10.6) |
| Stabilized | 92 (55.4) | 254 (42.8) |
| Up-titrated | 23 (13.9) | 46 (7.7) |
| **Japan** | *n* = 143 | *n* = 487 |
| Discontinued | 14 (9.8) | 191 (39.2) |
| Down-titrated | 9 (6.3) | 38 (7.8) |
| Stabilized | 102 (71.3) | 238 (48.9) |
| Up-titrated | 18 (12.6) | 20 (4.1) |

Values are presented as *n* (%). The sensitivity analysis was performed such that the definition of RAASi did not account for MRA. Patients with no other RAASi class at baseline were excluded.
CKD, chronic kidney disease; HF, heart failure; K^+^, potassium; MRA, mineralocorticoid receptor antagonist; RAASi, renin–angiotensin–aldosterone system inhibitor; SZC, sodium zirconium cyclosilicate.

Supplementary Table S4: Patient characteristics of the propensity score–matched SZC and no K^+^ binder cohorts at baseline: CKD subgroup (USA and Japan).

| Characteristic | USA | | Japan | |
| --- | --- | --- | --- | --- |
|  | SZC (*n* = 530) | No K^+^ binder (*n* = 1937) | SZC (*n* = 211) | No K^+^ binder (*n* = 711) |
| Age, years, mean ± SD | 72.3 ± 10.0 | 72.6 ± 9.9 | 73.8 ± 12.2 | 73.7 ± 12.8 |
| Sex, female, *n* (%) | 234 (44.2) | 858 (44.3) | 64 (30.3) | 208 (29.3) |
| HK severity at index, *n* (%)^a,b^ |  |  |  |  |
| Mild | 28 (27.2) | 111 (30) | 3 (18.8) | 8 (25.0) |
| Moderate | 45 (43.7) | 150 (40.5) | 8 (50.0) | 15 (46.9) |
| Severe | 30 (29.1) | 109 (29.5) | 5 (31.3) | 9 (28.1) |
| Missing | 427 (80.6) | 1567 (80.9) | 195 (92.4) | 679 (95.5) |
| CKD stage, *n* (%) |  |  |  |  |
| CKD3 | 249 (47.0) | 907 (46.8) | 37 (17.5) | 107 (15.1) |
| CKD4 | 223 (42.1) | 835 (43.1) | 73 (34.6) | 268 (37.7) |
| CKD5 | 58 (10.9) | 195 (10.1) | 101 (47.9) | 336 (47.3) |
| Heart failure, *n* (%) | 176 (33.2) | 660 (34.1) | 157 (74.4) | 524 (73.70) |
| Diabetes, *n* (%) | 420 (79.3) | 1543 (79.7) | 211 (100) | 711 (100) |
| Recurrent or new onset HK, n (%) |  |  |  |  |
| New onset | 38 (7.2) | 125 (6.5) | 26 (12.3) | 30 (4.2) |
| Recurrent | 492 (92.8) | 1812 (93.6) | 185 (87.7) | 681 (95.8) |
| K^+^ binder prescription before index, *n* (%) | 290 (54.7) | 973 (50.2) | 169 (80.1) | 629 (88.5) |

^a^Proportions are calculated excluding missing data.
^b^Mild, moderate and severe HK equate to K^+^ values of 5–5.49, 5.5–5.99, and ≥6.0 mmol/l, respectively.
CKD, chronic kidney disease; HK, hyperkalaemia; K^+^, potassium; SD, standard deviation; SZC, sodium zirconium cyclosilicate.

Supplementary Table S5: Patient characteristics of the propensity score–matched SZC and no K^+^ binder cohorts at baseline: HF subgroup (USA and Japan).

| Characteristic | USA | | Japan | |
| --- | --- | --- | --- | --- |
|  | SZC (*n* = 193) | No K^+^ binder (*n* = 703) | SZC (*n* = 571) | No K^+^ binder (*n* = 1887) |
| Age, years, mean ± SD | 73.4 ± 9.1 | 73.3 ± 9.0 | 77.1 ± 10.7 | 76.9 ± 10.3 |
| Sex, female, *n* (%) | 90 (46.6) | 332 (47.2) | 193 (33.8) | 627 (33.2) |
| HK severity at index, *n* (%)^a,b^ |  |  |  |  |
| Mild | 10 (27.0) | 38 (27.1) | 18 (32.1) | 57 (45.2) |
| Moderate | 13 (35.1) | 49 (35.0) | 23 (41.1) | 41 (32.5) |
| Severe | 14 (37.8) | 53 (37.9) | 15 (26.8) | 28 (22.2) |
| Missing | 156 (80.8) | 563 (80.1) | 515 (90.2) | 1761 (93.3) |
| CKD, *n* (%) | 176 (91.2) | 648 (92.2) | 430 (75.3) | 1512 (80.1) |
| CKD stage, *n* (%)^a^ |  |  |  |  |
| CKD3 | 71 (41.5) | 264 (41.1) | 25 (16.3) | 71 (14.1) |
| CKD4 | 78 (45.6) | 306 (47.6) | 51 (33.3) | 175 (34.7) |
| CKD5 | 22 (12.9) | 73 (11.4) | 77 (50.3) | 258 (51.2) |
| Missing | 8 (4.2) | 20 (2.8) | 277 (48.5) | 1008 (53.4) |
| Diabetes, *n* (%) | 158 (81.9) | 572 (81.4) | 520 (91.1) | 1736 (92.0) |
| Recurrent or new onset HK, n (%) |  |  |  |  |
| New onset | 10 (5.2) | 37 (5.3) | 122 (21.4) | 210 (11.1) |
| Recurrent | 183 (94.8) | 666 (94.7) | 449 (78.6) | 1677 (88.9) |
| K^+^ binder prescription before index, *n* (%) | 103 (53.4) | 348 (49.5) | 405 (70.9) | 1534 (81.3) |

^a^Proportions are calculated excluding missing data.
^b^Mild, moderate, and severe HK equate to K^+^ values of 5–5.49, 5.5–5.99, and ≥6.0 mmol/l, respectively.
CKD, chronic kidney disease; HF, heart failure; HK, hyperkalaemia; K^+^, potassium; SD, standard deviation; SZC, sodium zirconium cyclosilicate.

Supplementary Table S6: Patient characteristics of the propensity score–matched SZC and no K^+^ binder cohorts at baseline: CKD + HF subgroup (USA and Japan).

| Characteristic | USA | | Japan | |
| --- | --- | --- | --- | --- |
|  | SZC (*n* = 173) | No K^+^ binder (*n* = 635) | SZC (*n* = 148) | No K^+^ binder (*n* = 510) |
| Age, years, mean ± SD | 73.6 ± 9.3 | 74.0 ± 8.9 | 74.3 ± 13.1 | 74.2 ± 13.5 |
| Sex, female, *n* (%) | 82 (47.4) | 308 (48.5) | 49 (33.1) | 165 (32.4) |
| HK severity at index, *n* (%)^a,b^ |  |  |  |  |
| Mild | 9 (27.3) | 30 (25.2) | 3 (21.4) | 9 (36.0) |
| Moderate | 12 (36.4) | 45 (37.8) | 6 (42.9) | 10 (40.0) |
| Severe | 12 (36.4) | 44 (37.0) | 5 (35.7) | 6 (24.0) |
| Missing | 140 (80.9) | 516 (81.3) | 134 (90.5) | 485 (95.1) |
| CKD stage, *n* (%) |  |  |  |  |
| CKD3 | 69 (39.9) | 248 (39.1) | 22 (14.9) | 63 (12.4) |
| CKD4 | 81 (46.8) | 303 (47.7) | 48 (32.4) | 159 (31.2) |
| CKD5 | 23 (13.3) | 84 (13.2) | 78 (52.7) | 288 (56.5) |
| Diabetes, *n* (%) | 145 (83.8) | 509 (80.2) | 148 (100) | 510 (100) |
| Recurrent or new onset HK, n (%) |  |  |  |  |
| New onset | 7 (4.1) | 24 (3.8) | 12 (8.1) | 17 (3.3) |
| Recurrent | 166 (96.0) | 611 (96.2) | 136 (91.9) | 493 (96.7) |
| K^+^ binder prescription before index, *n* (%) | 95 (54.9) | 334 (52.6) | 122 (82.4) | 433 (84.9) |

^a^Proportions are calculated excluding missing data.
^b^Mild, moderate and severe HK equate to K^+^ values of 5–5.49, 5.5–5.99, and ≥6.0 mmol/l, respectively.
CKD, chronic kidney disease; HF, heart failure; HK, hyperkalaemia; K^+^, potassium; SD, standard deviation; SZC, sodium zirconium cyclosilicate.

Supplementary Table S7: Proportions of patients who discontinued, down‑titrated, stabilized and up-titrated their RAASi therapy: CKD subgroup (USA and Japan).

| CKD | SZC | No K^+^ binder | *P*-value |
| --- | --- | --- | --- |
| **USA** | *n* = 530 | *n* = 1937 |  |
| Discontinued | 103 (19.4) | 662 (34.2) | < .0001 |
| Down-titrated | 57 (10.8) | 237 (12.2) | .3916 |
| Stabilized | 309 (58.3) | 915 (47.2) | < .0001 |
| Up-titrated | 61 (11.5) | 123 (6.4) | < .0001 |
| **Japan** | *n* = 211 | *n* = 711 |  |
| Discontinued | 24 (11.4) | 286 (40.2) | < .0001 |
| Down-titrated | 19 (9.0) | 51 (7.2) | .4629 |
| Stabilized | 143 (67.8) | 315 (44.3) | < .0001 |
| Up-titrated | 25 (11.9) | 59 (8.3) | .1506 |

Values are presented as *n* (%). *P*-values for differences between the SZC cohort versus the no K^+^ binder cohort in the proportions of patients who discontinued, down-titrated, stabilized and up-titrated their RAASi therapy at 180 days post-index versus pre-index were calculated from χ^2^ tests.
CKD, chronic kidney disease; K^+^, potassium; RAASi, renin–angiotensin–aldosterone system inhibitor; SZC, sodium zirconium cyclosilicate.

Supplementary Table S8: Proportions of patients who discontinued, down‑titrated, stabilized and up-titrated their RAASi therapy: HF subgroup (USA and Japan).

| HF | SZC | No K^+^ binder | *P*-value |
| --- | --- | --- | --- |
| **USA** | *n* = 193 | *n* = 703 |  |
| Discontinued | 41 (21.2) | 241 (34.3) | .0008 |
| Down-titrated | 23 (11.9) | 131 (18.6) | .0372 |
| Stabilized | 104 (53.9) | 285 (40.5) | .0012 |
| Up-titrated | 25 (13.0) | 46 (6.5) | .0056 |
| **Japan** | *n* = 571 | *n* = 1887 |  |
| Discontinued | 55 (9.6) | 629 (33.3) | < .0001 |
| Down-titrated | 74 (13.0) | 196 (10.4) | .0997 |
| Stabilized | 370 (64.8) | 908 (48.1) | < .0001 |
| Up-titrated | 72 (12.6) | 154 (8.2) | .0017 |

Values are presented as *n* (%). *P*-values for differences between the SZC cohort versus the no K^+^ binder cohort in the proportions of patients who discontinued, down-titrated, stabilized and up-titrated their RAASi therapy at 180 days post-index versus pre-index were calculated from χ^2^ tests.
HF, heart failure; K^+^, potassium; RAASi, renin–angiotensin–aldosterone system inhibitor; SZC, sodium zirconium cyclosilicate.

Supplementary Table S9: Proportions of patients who discontinued, down-titrated, stabilized and up-titrated their RAASi therapy: CKD + HF subgroup (USA and Japan).

| CKD + HF | SZC | No K^+^ binder | *P*-value |
| --- | --- | --- | --- |
| **USA** | *n* = 173 | *n* = 635 |  |
| Discontinued | 39 (22.5) | 241 (38.0) | .0002 |
| Down-titrated | 17 (9.8) | 99 (15.6) | .0727 |
| Stabilized | 95 (54.9) | 248 (39.1) | .0003 |
| Up-titrated | 22 (12.7) | 47 (7.4) | .0390 |
| **Japan** | *n* = 148 | *n* = 510 |  |
| Discontinued | 15 (10.1) | 194 (38.0) | < .0001 |
| Down-titrated | 18 (12.2) | 68 (13.3) | .8153 |
| Stabilized | 94 (63.5) | 216 (42.4) | < .0001 |
| Up-titrated | 21 (14.2) | 32 (6.3) | .0032 |

Values are presented as *n* (%). *P*-values for differences between the SZC cohort versus the no K^+^ binder cohort in the proportions of patients who discontinued, down-titrated, stabilized and up-titrated their RAASi therapy at 180 days post-index versus pre-index were calculated from χ^2^ tests.
CKD, chronic kidney disease; HF, heart failure; K^+^, potassium; RAASi, renin–angiotensin–aldosterone system inhibitor; SZC, sodium zirconium cyclosilicate.

Supplementary Figure S1: Patient attrition flow-charts: A) the USA, B) Japan and C) Spain.

**A)**

**
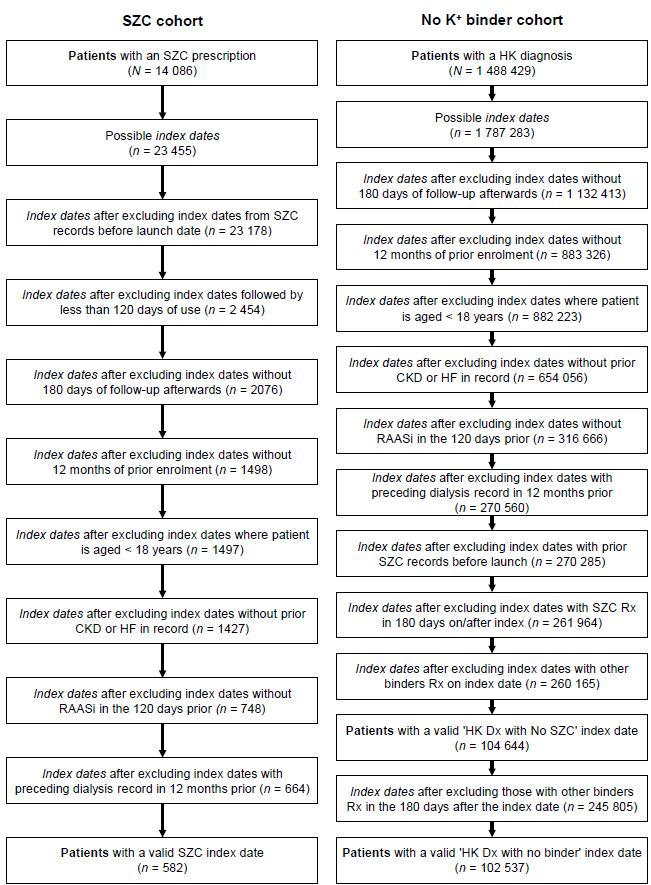
**

**B)**

**
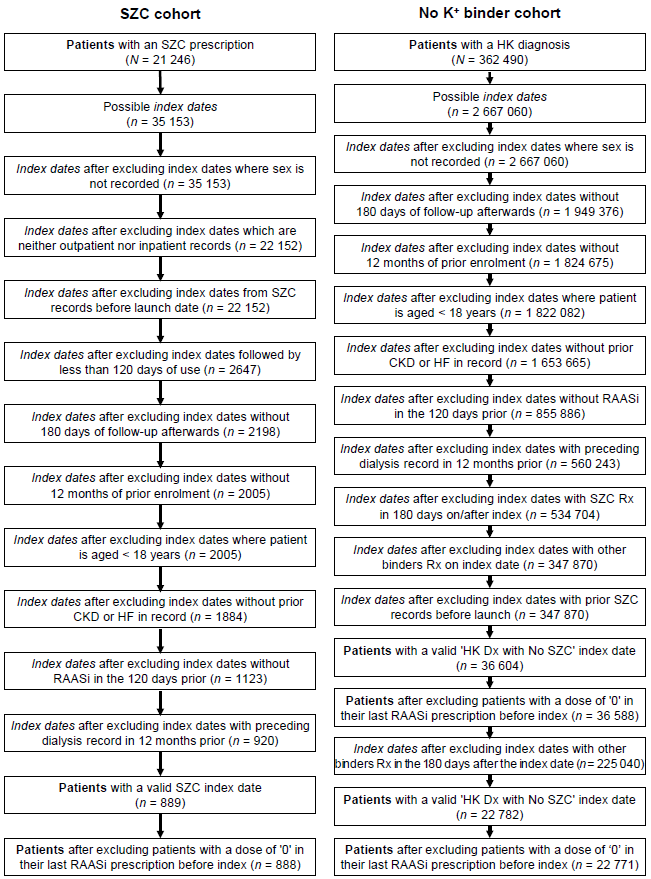
**

**C)**

**
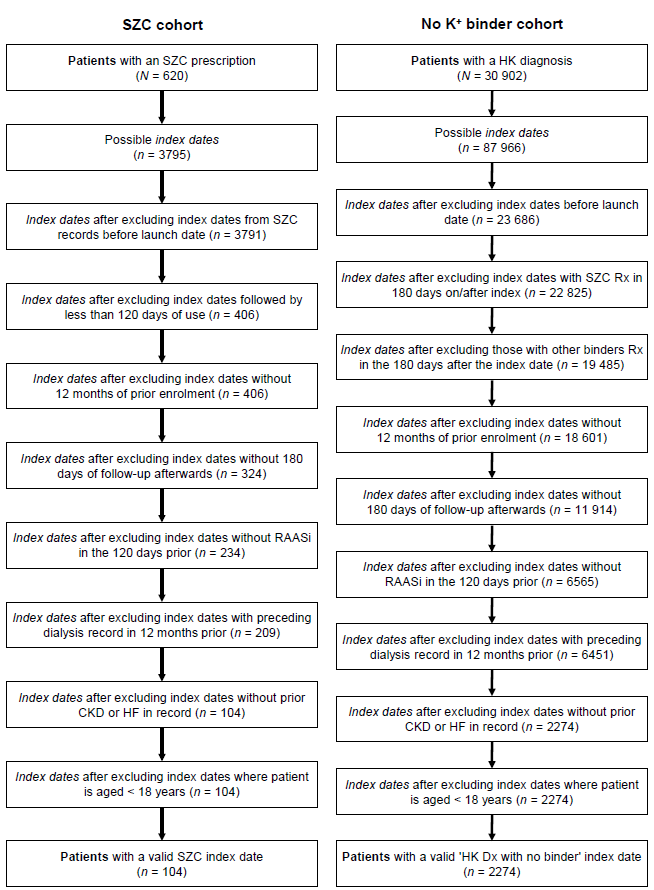
**

CKD, chronic kidney disease; Dx, diagnosis; HF, heart failure; HK, hyperkalaemia; K^+^, potassium; RAASi, renin–angiotensin–aldosterone system inhibitor; Rx, prescription; SZC, sodium zirconium cyclosilicate.

Supplementary Figure S2: Standardized mean difference plots of covariate balance before and after propensity score matching: A) the USA, B) Japan and C) Spain.

**A)**

**
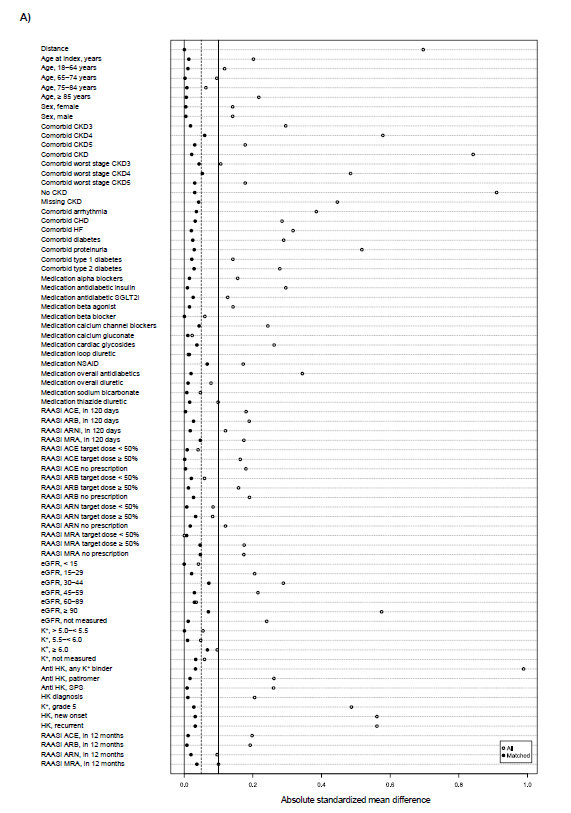
**

**B)**

**
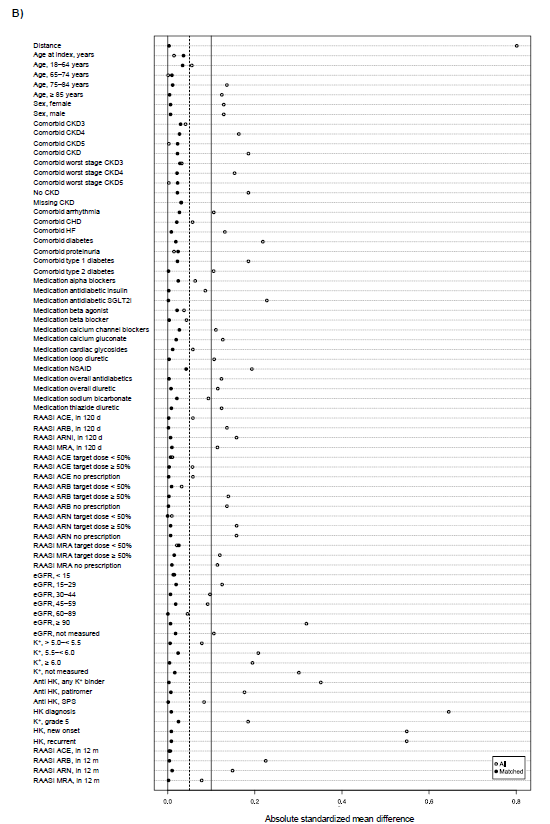
**

**C)**

**
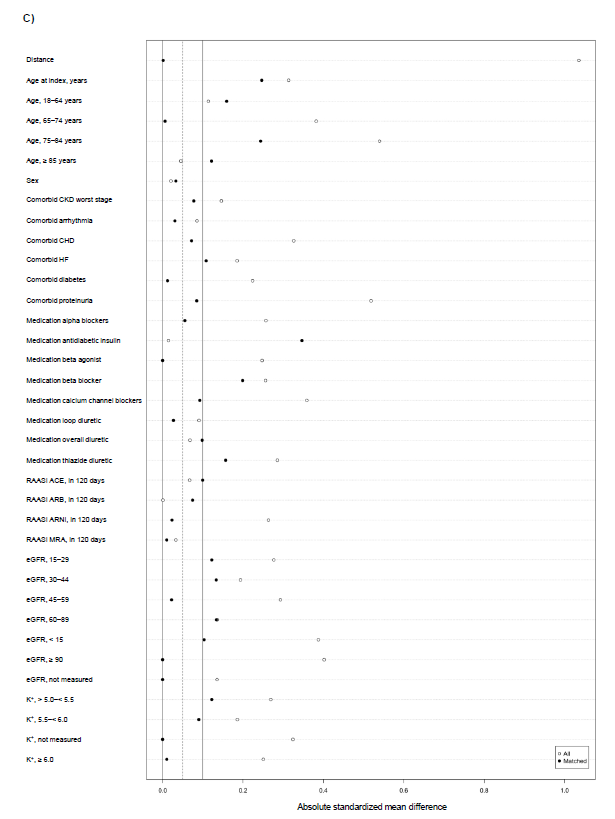
**

ACEi, angiotensin-converting enzyme inhibitor; ARB, angiotensin receptor blocker; ARNi, angiotensin receptor-neprilysin inhibitor; CHD, coronary heart disease; CKD, chronic kidney disease; eGFR, estimated glomerular filtration rate; HF, heart failure; HK, hyperkalaemia; K^+^, potassium; MRA, mineralocorticoid receptor antagonist; NSAID, non-steroidal anti-inflammatory drug; RAASi, renin–angiotensin–aldosterone system inhibitor; SGLT2i, sodium-glucose transport protein 2 inhibitor; SZC, sodium zirconium cyclosilicate.

Supplementary Figure S3: Distribution of propensity score before and after matching: A) the USA, B) Japan and C) Spain.

**
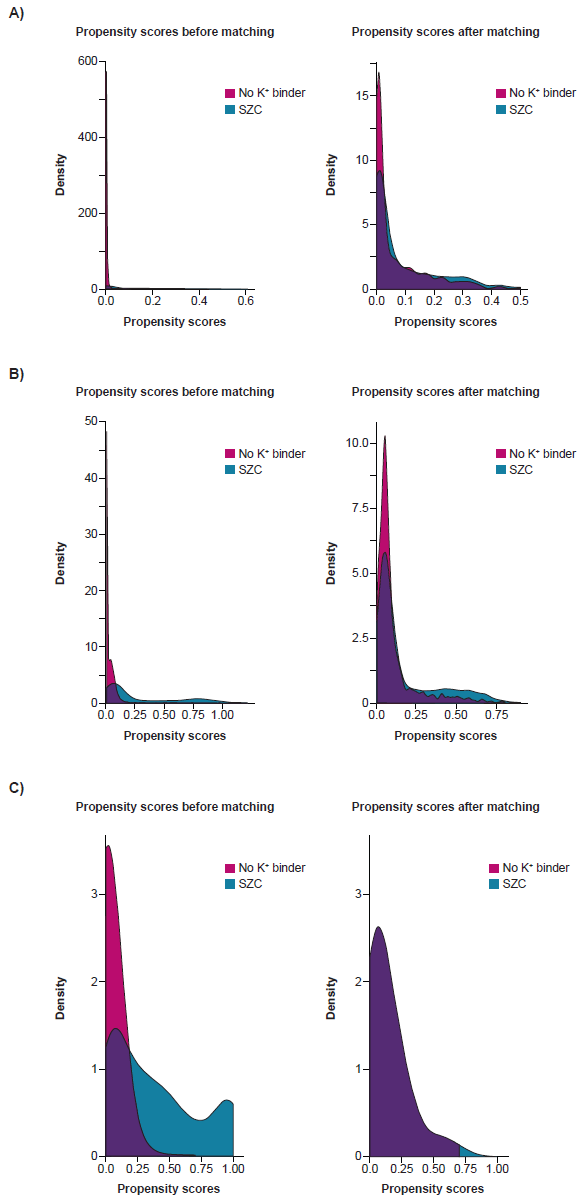
**

K^+^, potassium; SZC, sodium zirconium cyclosilicate.
